# Spinally projecting preproglucagon axons preferentially innervate sympathetic preganglionic neurons

**DOI:** 10.1016/j.neuroscience.2014.10.043

**Published:** 2015-01-22

**Authors:** I.J. Llewellyn-Smith, N. Marina, R.N. Manton, F. Reimann, F.M. Gribble, S. Trapp

**Affiliations:** a Cardiovascular Medicine, Physiology and Centre for Neuroscience, Flinders University, Bedford Park, SA 5042, Australia; b Department of Metabolism and Experimental Therapeutics, University College London, London WC1E, UK; c Department of Surgery and Cancer & Cell Biology Section, South Kensington Campus, Imperial College, London SW7 2AZ, UK; d Metabolic Research Laboratories, Wellcome Trust-MRC Institute of Metabolic Science, University of Cambridge, Addenbrooke’s Hospital, Hills Road, Cambridge CB2 0QQ, UK; e Department of Neuroscience, Physiology & Pharmacology, University College London, London WC1E 6BT, UK

**Keywords:** AP, area postrema, CAA, central autonomic area, ChAT, choline acetyltransferase, DAB, diaminobenzidine, DMH, dorsomedial nucleus of the hypothalamus, DMV, dorsal motor nucleus of the vagus, Ex-4, exendin-4, FG, Fluorogold, GFP, green fluorescent protein, GLP-1, glucagon-like peptide 1, ICN, intercalated nucleus, IML, intermediolateral cell column, IRT, intermediate reticular nucleus, L, lumbar, LE, lumbar enlargement, NHS, normal horse serum, NOS, nitric oxide synthase, NTS, nucleus of the solitary tract, PPG, preproglucagon, PVN, paraventricular nucleus of the hypothalamus, S, sacral, SPN, sympathetic preganglionic neuron, T, thoracic, YFP, yellow fluorescent protein, choline acetyltransferase, green fluorescent protein, glucagon-like peptide-1, nucleus of the solitary tract, parasympathetic preganglionic neurons, retrograde tracing

## Abstract

Glucagon-like peptide-1 (GLP-1) affects central autonomic neurons, including those controlling the cardiovascular system, thermogenesis, and energy balance. Preproglucagon (PPG) neurons, located mainly in the nucleus tractus solitarius (NTS) and medullary reticular formation, produce GLP-1. In transgenic mice expressing glucagon promoter-driven yellow fluorescent protein (YFP), these brainstem PPG neurons project to many central autonomic regions where GLP-1 receptors are expressed. The spinal cord also contains GLP-1 receptor mRNA but the distribution of spinal PPG axons is unknown.

Here, we used two-color immunoperoxidase labeling to examine PPG innervation of spinal segments T1–S4 in YFP-PPG mice. Immunoreactivity for YFP identified spinal PPG axons and perikarya. We classified spinal neurons receiving PPG input by immunoreactivity for choline acetyltransferase (ChAT), nitric oxide synthase (NOS) and/or Fluorogold (FG) retrogradely transported from the peritoneal cavity. FG microinjected at T9 defined cell bodies that supplied spinal PPG innervation.

The deep dorsal horn of lower lumbar cord contained YFP-immunoreactive neurons. Non-varicose, YFP-immunoreactive axons were prominent in the lateral funiculus, ventral white commissure and around the ventral median fissure. In T1–L2, varicose, YFP-containing axons closely apposed many ChAT-immunoreactive sympathetic preganglionic neurons (SPN) in the intermediolateral cell column (IML) and dorsal lamina X. In the sacral parasympathetic nucleus, about 10% of ChAT-immunoreactive preganglionic neurons received YFP appositions, as did occasional ChAT-positive motor neurons throughout the rostrocaudal extent of the ventral horn. YFP appositions also occurred on NOS-immunoreactive spinal interneurons and on spinal YFP-immunoreactive neurons. Injecting FG at T9 retrogradely labeled many YFP-PPG cell bodies in the medulla but none of the spinal YFP-immunoreactive neurons.

These results show that brainstem PPG neurons innervate spinal autonomic and somatic motor neurons. The distributions of spinal PPG axons and spinal GLP-1 receptors correlate well. SPN receive the densest PPG innervation. Brainstem PPG neurons could directly modulate sympathetic outflow through their spinal inputs to SPN or interneurons.

## Highlights

• Spinal GLP-1 axons target primarily sympathetic preganglionic neurons.
• Spinal GLP-1 axons innervate interneurons that may regulate sympathetic outflow.
• Many GLP-1 neurons in the medulla are spinally-projecting.
• The lumbar cord contains YFP-expressing neurons that do not innervate the brain.

## Introduction

Glucagon-like peptide 1 (GLP-1) is released post-prandially from enteroendocrine cells in the gut and facilitates absorption of nutrients (Holst, 2007). GLP-1 is also a potent satiety signal (Turton et al., 1996) and treatment with GLP-1 analogs results in sustained weight loss in humans (Drucker and Nauck, 2006; Buse et al., 2009; Holst, 2013). Furthermore, studies using microinjections of GLP-1 receptor antagonists into specific brain regions in rodents indicate that, within the central nervous system (CNS), endogenous GLP-1 can also produce satiety (Hayes et al., 2009; Williams et al., 2009; Barrera et al., 2011; Dossat et al., 2011; Alhadeff et al., 2012).

In the CNS preproglucagon (PPG) is processed post-translationally to produce GLP-1, GLP-2, and oxyntomodulin (Holst, 2007). The highest levels of these PPG products occur in the dorsomedial hypothalamus (DMH) and hypothalamic paraventricular nucleus (PVN) and the lowest levels are found in the cortex and hindbrain (Jin et al., 1988; Vrang et al., 2007; Tauchi et al., 2008). The medulla oblongata contains most of the brain’s GLP-1-synthesizing neurons. The majority of GLP-1-producing somata in the brainstem occur in the caudal nucleus tractus solitarius (NTS). Some GLP-1 cell bodies are also located in the dorsomedial region of the medullary reticular nucleus (Jin et al., 1988; Larsen et al., 1997). Retrograde tracing has verified that the GLP-1 axons in the hypothalamus arise from these two subgroups of GLP-1 neurons (Larsen et al., 1997; Vrang et al., 2007). Consistent with these immunocytochemical data, the only brain regions that contain detectable levels of PPG mRNA are the caudal NTS, the intermediate reticular nucleus (IRT) and the olfactory bulb (Merchenthaler et al., 1999). Using transgenic mice in which expression of yellow fluorescent protein (YFP) is controlled by the glucagon promoter (Reimann et al., 2008), we have previously described the full distribution of PPG cell bodies and dendrites within the medulla and shown widespread GLP-1 innervation throughout the brain (Llewellyn-Smith et al., 2011). This distribution of YFP-PPG axons within the brain parallels the distribution of brain GLP-1 receptors (Shughrue et al., 1996; Merchenthaler et al., 1999). Taken together, these anatomical findings suggest that GLP-1 release from PPG axons within the brain could influence homeostatic responses and help to coordinate energy balance, food intake and cardiovascular function.

More recently, we have demonstrated that PPG neurons directly innervate cranial parasympathetic preganglionic neurons in the dorsal motor nucleus of the vagus (DMV), again using YFP-PPG mice (Llewellyn-Smith et al., 2013). We also showed that populations of ventral medullary neurons that have been implicated in central sympathetic control received PPG input. Although the spinal cord has not yet been examined for the presence of PPG mRNA, perikarya expressing mRNA for GLP-1 receptors occur in laminae V–X throughout the rat spinal cord (Merchenthaler et al., 1999). Hence, our observations raised the possibility that GLP-1 neurons in the brainstem might also provide direct input to spinal autonomic neurons.

Here, we demonstrate that there is GLP-1 innervation of neurons in the spinal cord that contain immunoreactivity for the enzymes that synthesize acetylcholine and nitric oxide. As in previous work (Hisadome et al., 2010; Llewellyn-Smith et al., 2011, 2013), we used transgenic YFP-PPG mice in order to take advantage of the strong YFP expression that occurs throughout the cytoplasm of PPG neurons, including their terminals. We detected spinal PPG axons by the presence of YFP-immunoreactivity and identified their innervation targets using immunoreactivity for choline acetyltransferase (ChAT), nitric oxide synthase (NOS), and/or Fluorogold (FG) retrogradely transported from the peritoneal cavity. We determined the source of the spinal GLP-1 innervation by retrograde transport of FG from injections into thoracic (T) spinal segment T9.

## Experimental procedures

All experiments were carried out in accordance with the UK Animals (Scientific Procedures) Act, 1986 and had the required ethics approvals. We used a total of 21 adult mGLU-124 Venus YFP mice (Reimann et al., 2008), which we call YFP-PPG mice here. Bred at Imperial College, the 13 male and eight female mice received food and water *ad libitum* and were kept on a 12-h light:12-h dark cycle. When perfused at 12–16 weeks after birth, the mice weighed 25–35 g, with females being lighter than males of the same age.

### Retrograde tracing with FG

#### Intraperitoneal injections

Three male YFP-PPG mice had FG (0.2% in distilled water, 40 μl) injected into the peritoneal cavity as in Anderson and Edwards (1994).

#### Spinal injections

Three male and two female YFP-PPG mice were anesthetized with ketamine (75 mg/kg; i.m.) and medetomidine (0.3 mg/kg; i.m.) and placed in a stereotaxic frame. The thoracic spinal cord (segment T9) was carefully exposed by retracting the intervertebral space between vertebrae T6–T7 followed by removal of the yellow ligament. The dura mater was pierced with a 19-g needle and the intermediolateral cell column (IML) was unilaterally targeted with a microinjection of FG (2%, 50 nl) placed 0.2 mm lateral to the midline and 0.5 mm ventral from the dorsal surface of the spinal cord. Anesthesia was reversed with atipamezol (1 mg/kg; i.m.) and postoperative analgesia was given for 4 days (buprenorphine, 1 mg/kg). Mice recovered normally without abnormalities in locomotion. All mice with FG injections were perfused transcardially 7 days after surgery. Correct targeting of the area with FG and its spread within the spinal cord was confirmed postmortem by analysis of spinal cord sections immunoperoxidase stained for FG.

### Perfusion and tissue preparation

YFP-PPG mice under ketamine and medetomidine anesthesia were heparinized (500 IU/l) and their blood was removed with a flush of phosphate-buffered saline. The mice were then transcardially perfused with 60 ml of phosphate-buffered 4% formaldehyde. After 3 days of post-fixation, brains and spinal cords still in their vertebral columns were shipped to Flinders for sectioning and immunohistochemical processing.

Spinal cords were removed from their vertebral columns, post-fixed at room temperature on a shaker for 2–3 days in phosphate-buffered 4% formaldehyde and then divided into segments. The rostral edge of the dorsal root entry zone was considered to mark the rostral boundary of each segment. For horizontal sections, cords from two male YFP-PPG mice were divided into three lengths (T1–T7, T8–L3 and L4–S4). For transverse sectioning, thoracic and upper lumbar cords from 19 mice were divided into T1–3 and then two-segment length blocks through to segment L3; segments L4–S4 were left as one piece. The blocks containing T1–L3 from each mouse were embedded together in albumin gelatine (Llewellyn-Smith et al., 2007) to form a single block. Segments L4–S4 from two to three mice were embedded together in a single block of albumin gelatine. The albumin gelatine blocks for transverse sectioning and the cord lengths for horizontal sectioning were infiltrated with 20% then 30% sucrose. Blocks for transverse sectioning were cut at 25 μm on a cryostat. Cord segments for horizontal cryostat sections were cut at 30 μm.

The medullas of mice with injections of FG at T9 were blocked without the use of a brain matrix, infiltrated with sucrose and cut into three series of transverse 30-μm cryostat sections. Because of variability in dorsoventral tilt between blocks, we could not assign Bregma values to brainstem sections.

### Immunohistochemistry

Cryostat sections were first washed 3 × 10 min in 10 mM Tris, 0.9% NaCl, 0.05% thimerosal in 10 mM phosphate buffer, pH 7.4, (TPBS) containing 0.3% Triton X-100 for removal of membranes and then exposed to TPBS-Triton containing 10% normal horse serum (NHS) for at least 30 min to block non-specific antibody binding. Primary antibodies were diluted with TPBS-Triton containing 10% NHS; secondary antibodies, with TPBS-Triton containing 1% NHS; and the avidin-horseradish peroxidase complex, with TPBS-Triton. All steps took place at room temperature on a shaker and sections were washed 3 × 10 min in TPBS after each incubation in an immunoreagent.

The optimal dilutions of primary antibodies (Table 1) were determined by titration and gave the maximum number of immunoreactive structures with minimal non-specific background staining. For optimally visualizing the spinal axons of YFP-expressing neurons, the anti-green fluorescent protein (GFP) antibody was used more dilute than for optimally visualizing their cell bodies (see Llewellyn-Smith et al., 2011).

#### Immunoperoxidase labeling

All antigens were localized with a standard avidin–biotin-peroxidase protocol and either black or brown diaminobenzidine (DAB) reaction products. The sections that had been washed in TPBS-Triton and exposed to 10% NHS as above were transferred into the first primary antibody for 2–5 days. After washing, sections were incubated overnight in a biotinylated donkey anti-immunoglobulin (Ig) antibody (1:500; Jackson ImmunoResearch, West Grove PA) followed by a 4–6-h incubation in ExtrAvidin-peroxidase (1:1500; Sigma–Aldrich, St Louis, MO, USA). A cobalt + nickel-intensified DAB reaction in which peroxide was generated by glucose oxidase (Llewellyn-Smith et al., 2005) stained structures immunoreactive for the first antigen black. After the first antigen had been localized with a black DAB reaction product, the sections were washed and underwent another blocking step in NHS. Then the immunohistochemical protocol above was repeated to localize the second antigen, which was stained brown with an imidazole-intensified DAB reaction with peroxide again produced by a glucose oxidase reaction (Llewellyn-Smith et al., 2005).

Localization of the following combinations of antigens revealed the innervation targets of spinal PPG axons: YFP (black) + ChAT (brown); YFP (black) + NOS (brown); YFP (black) + FG (black) + NOS (brown). Immunostaining for FG (black) and YFP (brown) defined whether YFP-immunoreactive cell bodies in the dorsal horn of lower lumbar cord sent axons rostrally through FG injection sites in T9.

After immunoperoxidase staining, sections were mounted in serial order onto chrome alum-gelatine-coated slides, dried, cleared and coverslipped with Permaslip mounting medium (Alban Scientific, St Louis, MO, USA).

#### Immunofluorescence labeling

Double immunofluorescent labeling was performed on brainstem sections from two female and three male YFP-PPG mice with FG injections at T9. After washes in Tris-PBS-Triton and exposure to 10% NHS, the sections were incubated in anti-GFP (1:10,000) plus anti-FG (1:2000), then in FITC-donkey anti-chicken IgY (1:200) plus biotinylated donkey anti-guinea-pig IgG (1:500) and finally in Cy3-Streptavidin (1:1000; Amersham catalog# PA43001; GE Healthcare Bio-Sciences Pty. Ltd., Rydalmere, NSW, Australia). Incubation times were the same as in the immunoperoxidase protocol. Buffered glycerol was used to mount the immunofluorescently stained sections in serial order. Coverslips were applied and sealed with nail polish to prevent dehydration. Slides were stored in the dark at 4 °C until examination.

#### Antibody characterization

The chicken anti-GFP antiserum used here specifically recognizes YFP. In Llewellyn-Smith et al. (2011), we demonstrated that sections from animals lacking YFP-immunoreactivity showed no labeling when stained with this antibody. Furthermore, the antibody detected immunoreactivity in all of the brainstem neurons that showed native YFP fluorescence.

We have previously characterized the Chemicon goat anti-ChAT antiserum (Llewellyn-Smith et al., 2005, 2007). Absorption of a 1:2000 dilution of this antiserum with 2 μg/ml recombinant rat ChAT abolished staining in sections of similarly fixed and processed tissue. Furthermore, cholinergic neurons visualized with this anti-ChAT antiserum showed the expected spinal distribution (Fenwick et al., 2006).

We have also characterized the ImmunoStar rabbit anti-NOS antiserum (Hinrichs and Llewellyn-Smith, 2009). NOS immunoreactivity was not detectable when we stained sections of formaldehyde-fixed spinal cord with 1:50,000 anti-NOS antiserum that had been absorbed overnight with 0.5 μg/ml of control peptide.

The specificity of the Protos guinea-pig anti-FG antibody was also defined using overnight absorption (Llewellyn-Smith et al., 2007). A 1:20,000 dilution of the anti-FG antiserum preabsorbed with 10 μg/ml FG failed to stain sections containing neurons retrogradely labeled with FG.

### Data collection and analysis

We used an Olympus BH-2 brightfield microscope to examine immunoperoxidase-stained sections containing spinal segments T1–S4. Using a ×100 oil immersion lens, we determined whether close appositions from black YFP-immunoreactive axon terminals occurred on spinal neurons with brown immunoreactivity for ChAT or NOS, brown NOS-immunoreactivity plus black FG-immunoreactivity or black YFP-immunoreactivity. We classed YFP terminals lying side-by-side with an immunolabeled neuron as closely apposed when no space separated the terminal and the neuron. Where terminals crossed over immunoreactive neurons, an apposition was considered close when the neuron and the terminal were in the same focal plane.

Immunofluorescently stained brainstem sections from the caudal tip of the 4th ventricle to the spinomedullary junction were examined with an Olympus AX70 epifluorescence microscope fitted with a single-wavelength LED illumination system and emission filters that unequivocally distinguished red and green fluorophores without bleed-through. For FITC, the excitation wavelength was 490 nm; and the emission wavelengths, 515–545 nm. For Cy3, wavelengths were 525 nm for excitation and 575–615 nm for emission.

We used a SPOT Insight Model 18.2 firewire color camera and SPOT RT software version 4.6 (Diagnostic Instruments Inc., Sterling Heights, MI, USA) to collect digital images of immunoperoxidase-stained spinal neurons. Images of immunofluorescently stained brainstem neurons were captured with an Orca high-resolution cooled CCD digital camera (Hamamatsu, Hamamatsu City, Japan) and analySIS ruler software (version 5; Soft Imaging Solutions, an affiliate of Olympus, Olympus Soft Imaging Solutions GmbH, Münster, Germany). We used Adobe PhotoShop to adjust the sharpness, brightness, and contrast of images saved as TIFF files, to match the color of the digital images and to prepare montages and plates.

#### Counting spinal YFP-immunoreactive neurons that project rostrally

We determined whether YFP-immunoreactive neurons in the lumbar enlargement (LE) projected rostrally in 1:3 series of immunoperoxidase-stained transverse sections from two female YFP-PPG mice. We examined every section in the series and classed each brown YFP-immunoreactive soma as either positive or negative for the presence of black perinuclear puncta due to retrograde transport of FG from injections at T9.

#### Counting spinal neurons with YFP-immunoreactive appositions

*Parasympathetic preganglionic neurons (PPN):* We used our published counting strategy (Llewellyn-Smith et al., 2013) to determine the proportion of sacral PPN that received YFP-PPG innervation. Brown ChAT-immunoreactive PPN in the sacral parasympathetic nucleus were examined for close appositions provided by black YFP-immunoreactive axons in alternate 25-μm horizontal sections from two male YFP-PPG mice. The parasympathetic nucleus was photographed at ×10 magnification. In PhotoShop, the resulting TIFFS were montaged and two new layers were added to the montage. Using a ×100 oil immersion lens, we then inspected each PPN to determine whether any YFP-immunoreactive boutons apposed it. Using the pencil tool on one of the new PhotoShop layers, we marked the position of each neuron that received a YFP apposition with a blue dot. We marked the position of each neuron *without* any YFP appositions on the second layer with a red dot. To count PPN that did or did not receive close appositions from YFP-positive terminals, the layers with colored dots were saved as individual TIFF files. After opening each TIFF file in ImageJ (National Institutes of Health), we converted the image to black and white and used the Analyze Particle function to count dots.

*Spinal YFP-immunoreactive neurons:* Black YFP-immunoreactive neurons in the dorsal horn of the LE from seven mice were located in 1:3 or 1:6 series of sections stained for YFP plus ChAT and in 1:6 series of sections stained for YFP plus NOS. In each series of sections, we examined every YFP-expressing spinal neuron with a ×100 oil immersion objective to determine if it received close appositions (see criteria above) from YFP-immunoreactive terminals. In sections immunostained for YFP plus ChAT, spinal YFP-positive neurons were checked for the presence of close appositions from axons immunoreactive for ChAT. We assessed whether or not NOS-immunoreactive axons closely apposed YFP-labeled neurons in sections stained for YFP plus NOS.

#### Counting brainstem YFP-PPG neurons that project to the spinal cord

In one male mouse and two female mice with FG injections at T9, we examined green YFP-immunoreactive cell bodies in the NTS, IRT and midline for the presence of FG-immunoreactivity that had been retrogradely transported from the spinal cord. From each mouse, we collected digital images of all sections containing YFP-immunoreactive neurons from the NTS and IRT on one side of the brainstem or from the midline. Since medullas were sectioned into 1:3 series, 60 μm separated photographed sections. YFP-immunoreactive neurons were counted unilaterally in a total of 19 sections through the NTS and 18 sections through the IRT and in 13 sections through the midline.

To determine the proportion of YFP-PPG neurons that were spinally-projecting, we photographed green YFP-immunoreactive neurons in a region using a x10 objective and then photographed red FG-immunoreactive neurons in the same region. The two micrographs as TIFF files were false-colored appropriately and then overlaid in PhotoShop. Green YFP neurons were checked for the presence of red intracellular FG staining. The positions of neurons double-labeled for YFP and FG were marked with blue dots using the pencil tool on one new PhotoShop layer and the positions of neurons single-labeled for YFP were marked with magenta dots on a second new layer. To count YFP neurons with or without FG labeling, the two layers were saved as separate TIFF files, opened in ImageJ and converted to black and white. The Analyze Particle function was then used to count the dots. Data are presented as mean ± standard error (SEM).

## Results

### Native YFP fluorescence within the spinal cord

**Fig. 1:**
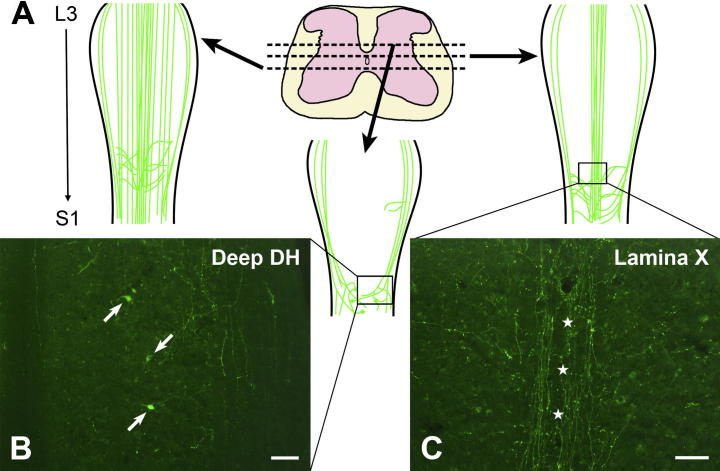
Distribution of spinal axons showing native YFP fluorescence in segments L3-S1. (A) Schematic showing the locations of YFP-fluorescent axons and cell bodies in longitudinal sections through the lower lumbar and upper sacral spinal cord at dorsoventral levels indicated in the diagram of the transverse section, which shows L3 (modified from the Allen Brain Institute Reference Atlas; http://mousespinal.brain-map.org/imageseries/showref.html). (B) In the lower lumbar cord, YFP-fluorescent nerve cell bodies (arrows) as well as YFP-fluorescent varicose and non-varicose axons occur within the deep dorsal horn (DH). (C) In dorsal lamina X, YFP-fluorescent axons travel rostrocaudally and mediolaterally. A rostrocaudal tract of varicose YFP-fluorescent axons (stars) lies just dorsal to the central canal. Scale bars: B and C = 100 μm.

The distribution of glucagon promoter-driven YFP expression in coronal and longitudinal sections of spinal cord between thoracic segment 1 (T1) and sacral segment 4 (S4) was assessed in seven adult YFP-PPG mice. ‘Native’ YFP-fluorescence was strong and both YFP-fluorescent axons and cell bodies could be identified without immunostaining (Fig. 1), as has been observed in brainstem (Hisadome et al., 2010; Llewellyn-Smith et al., 2011).

### YFP-immunoreactive cell bodies and dendrites within the spinal cord

**Fig. 2:**
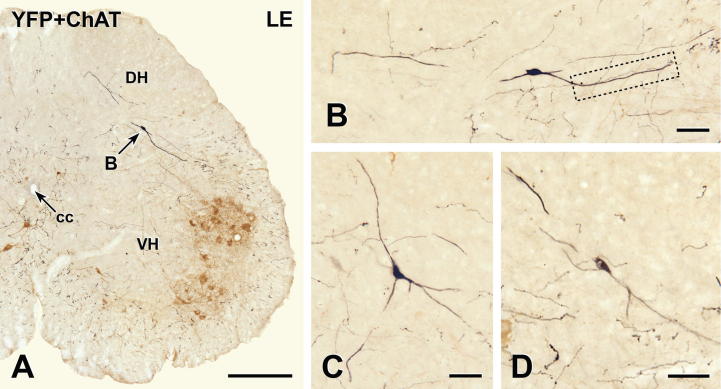
YFP-immunoreactive cell bodies and dendrites at the level of the lumbar enlargement (LE). Two-color immunoperoxidase staining for YFP (black) and ChAT (brown) in transverse sections through the lower lumbar spinal cord. (A) A YFP-immunoreactive cell body (black) in lamina V of the dorsal horn (DH) at spinal level L4–5. ChAT-immunoreactive somatic motor neurons (brown) occur in lamina IX in the ventral horn (VH). The arrow indicates the location of the neuron shown in B. cc, central canal. Montage of 18 micrographs. Scale bar = 250 μm. (B) Higher magnification image of the black YFP-immunoreactive neuron indicated by B in A. The boxed area is shown at higher magnification in Fig. 9A. Montage of 9 micrographs. (C and D) Other examples of black YFP-immunoreactive cell bodies and their dendrites in lamina V of the lumbar enlargement. (D) Montage of 2 micrographs. Scale bars in B–D, 50 μm. (For interpretation of the references to color in this figure legend, the reader is referred to the web version of this article.)

Although native YFP fluorescence (Fig. 1) revealed that YFP-expressing neurons existed in the lumbosacral spinal cord, an anti-GFP antibody with peroxidase detection was better for defining the morphology of the spinal YFP-positive neurons (Fig. 2). YFP-immunoreactive cell bodies were present in the deep dorsal horn (laminae IV and V) and were located mainly in the LE. The segmental distribution of YFP-positive spinal neurons never overlapped with the segmental distribution of sympathetic preganglionic neurons (SPN). However, rare YFP-expressing neurons occurred in the same sections as the most rostrally located PPN. The spinal YFP-immunoreactive neurons had fusiform or multipolar cell bodies and a small number of dendrites with few branches. The best-stained YFP-positive neurons had dendrites that extended for long distances both laterally and dorsally. The primary and secondary dendrites of YFP-immunoreactive neurons generally were smooth but a few intensely stained dendrites exhibited small dendritic spines. The somata of a number of the heavily stained YFP-expressing neurons gave rise to very fine processes that traveled for variable distances without changing diameter, a morphology that is suggestive of axons.

**Fig. 3:**
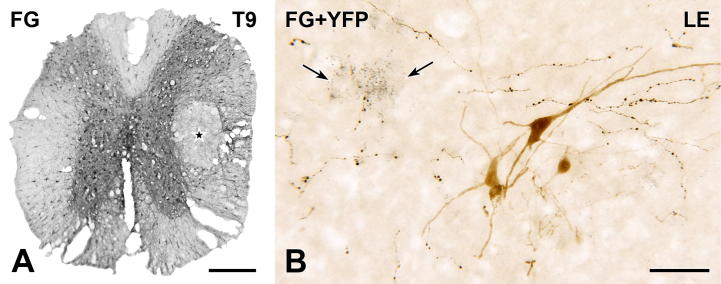
Spinal YFP-immunoreactive neurons do not project rostrally. (A) Transverse section single-stained to show FG-immunoreactivity at the center of an FG injection site (star) in thoracic segment T9. Peroxidase reaction product occurs throughout the spinal gray and white matter. Thus, it is highly likely that all axons passing through the injection site have taken up FG. Montage of 6 micrographs. Scale bar = 250 μm. (B) Two-color immunoperoxidase staining for FG (black) and YFP (brown). Four brown YFP-immunoreactive cell bodies in the lumbar enlargement (LE) lack black FG-immunoreactivity, indicating that they do not project rostrally through T9. In contrast, black puncta of FG-immunoreactivity are present in nearby cell bodies (arrows) and in black, varicose axons of undefined neurochemical phenotype. Montage of 3 micrographs. Scale bar = 50 μm. (For interpretation of the references to color in this figure legend, the reader is referred to the web version of this article.)

Spinal injections of the retrograde tracer FG in five mice and subsequent immunoperoxidase processing of cords to detect FG-immunoreactivity allowed us to assess whether spinal YFP-expressing neurons in the LE sent axons to the brain (Fig. 3). The FG injection sites (Fig. 3A) were centered on the IML between segments T8 and T10. FG-immunoreactivity spread throughout the spinal gray and white matter for one to two segments in both rostral and caudal directions. To quantify the proportion of spinal YFP-containing neurons that sent axons through the T9 injection sites, we used double immunoperoxidase staining to localize FG (black) and YFP (brown) in transverse sections from two mice (Fig. 3B) and counted neurons that were single-stained for YFP or double-stained for YFP + FG. We examined 55 YFP-immunoreactive neurons in one mouse and 43 in the other mouse. Although neurons retrogradely labeled with FG lay very near to the YFP-expressing neurons (Fig. 3B), not one of the 98 spinal YFP-immunoreactive neurons that we examined showed immunoreactivity for FG.

### Distribution of YFP-immunoreactive axons within the spinal cord

After immunoperoxidase staining of either transverse or horizontal sections of spinal cord, both non-varicose and varicose YFP-PPG axons were intensely stained for YFP.

**Fig. 4:**
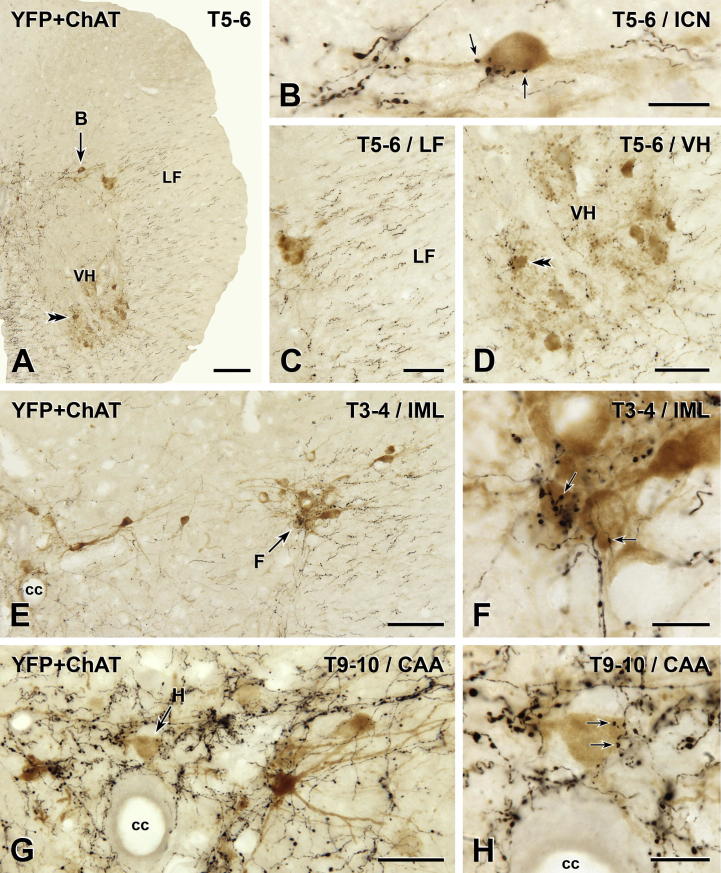
Distribution of YFP-PPG axons in thoracic spinal cord. Two-color immunoperoxidase staining for YFP (black) and ChAT (brown) in transverse sections through the thoracic spinal cord. (A–D) In spinal segments T5–6, there is a high density of rostrocaudally running black YFP-immunoreactive axons in the lateral funiculus (LF; C). There are close appositions between black YFP-immunoreactive varicosities and brown ChAT-immunoreactive sympathetic preganglionic neurons (SPN) in the intercalated nucleus (ICN; B) and brown ChAT-immunoreactive somatic motor neurons in the ventral horn (VH; D). A higher magnification image of the cell body indicated by the double arrowheads in A and D is shown in Fig. 7I. (E and F) Brown ChAT-immunoreactive SPN in the intermediolateral cell column (IML) of T3–4 receive close appositions (arrows) from black YFP-immunoreactive varicosities. (G and H) In the central autonomic area (CAA) at T9–10, black YFP-immunoreactive varicosities (black) also form appositions (arrows) on brown ChAT-immunoreactive SPN. cc, central canal. Micrographs in montages: A and E, 5; B, 6; C and D, 3; F & H, 2; G, 4. Scale bars: A and E, 100 μm; C, D and G, 50 μm; B, F and H, 20 μm. (For interpretation of the references to color in this figure legend, the reader is referred to the web version of this article.)

Many non-varicose YFP-immunoreactive axons traveled rostrocaudally in white matter tracts (Fig. 4). These axons were most dense in the ventral white commissure and around the ventral median fissure. Non-varicose axons were also common in the lateral and ventral funiculi in rostral thoracic segments. The density of non-varicose axons declined progressively from rostral to caudal segments.

**Fig. 5:**
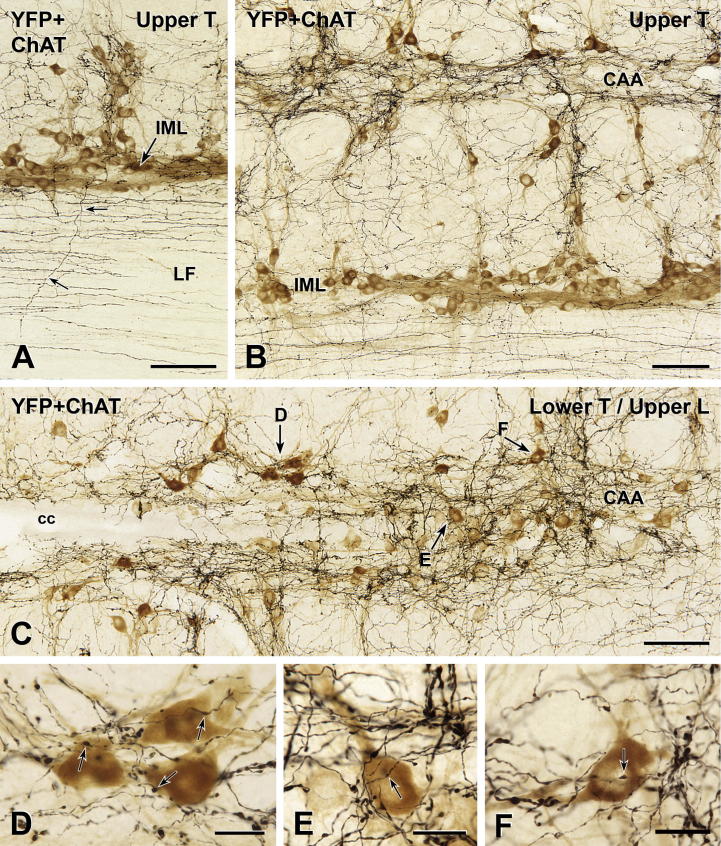
YFP-immunoreactive innervation of sympathetic preganglionic neurons (SPN). Two-color immunoperoxidase staining for YFP (black) and ChAT (brown) in horizontal sections through the spinal cord at upper thoracic (Upper T; A and B) and lower thoracic/upper lumbar (Lower T/Upper L; C) levels. At both spinal levels (B and C), black YFP-immunoreactive axons most densely innervate the central autonomic area (CAA), which lies dorsal to the central canal (cc) in lamina X. The intermediolateral cell column (IML) receives a moderately dense black YFP innervation. The lateral funiculus (LF; A and B) contains primarily black YFP-immunoreactive axons of passage that run rostrocaudally and occasional axons that travel mediolaterally (small arrows in A). The neurons indicated by arrows D–F are shown at higher magnification in D–F. Micrographs in montages: A, 3; B, 9; C, 6. (D–F) Black YFP-immunoreactive boutons from close appositions (arrows) on brown ChAT-immunoreactive SPN in the CAA. E, Montage of 2 micrographs. Scale bars: A–C, 100 μm; D–F, 20 μm. (For interpretation of the references to colour in this figure legend, the reader is referred to the web version of this article.)

**Fig. 6:**
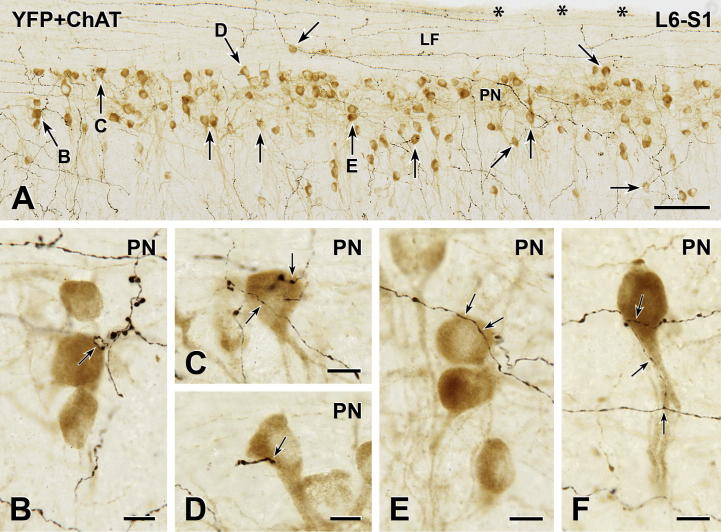
YFP-immunoreactive innervation of parasympathetic preganglionic neurons (PPN). Two-color immunoperoxidase staining for YFP (black) and ChAT (brown) in a horizontal section through the lumbosacral (L6-S1) spinal cord. (A) The sacral parasympathetic nucleus (PN) is sparsely innervated by black YFP-immunoreactive axons. Of the 133 PPN in A, black YFP-positive axon terminals closely appose only 29 of the brown ChAT-immunoreactive PPN, some of which are indicated by arrows. Arrows B-E mark neurons that are shown at higher magnification in B–E. Black, non-varicose YFP-immunoreactive axons run rostrocaudally through the white matter of the lateral funiculus (LF). Asterisks indicate the lateral edge of the spinal cord. Montage of 6 micrographs. Scale bar: 100 μm. (B–F) Brown ChAT-immunoreactive PPN receive close appositions (arrows) from black YFP-immunoreactive varicosities. Micrographs in montages: B, 4; C, 3; D and E, 2; F, 6. Scale bars: 10 μm. (For interpretation of the references to color in this figure legend, the reader is referred to the web version of this article.)

**Fig. 7:**
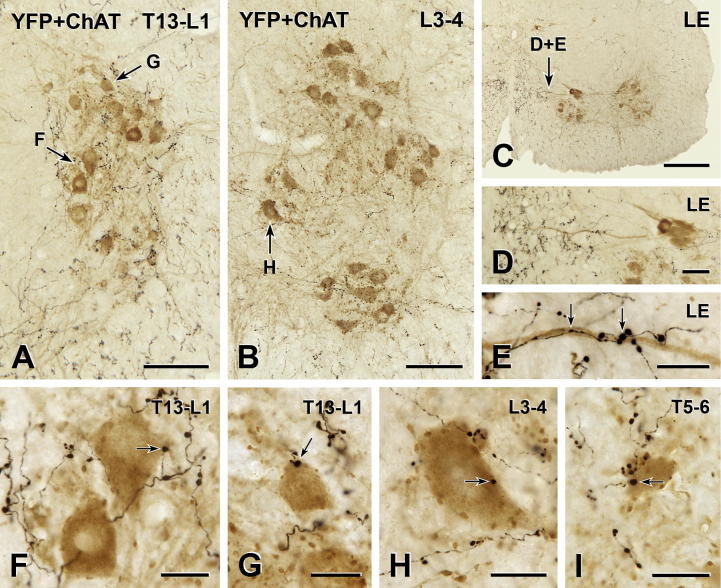
YFP-immunoreactive innervation of somatic motor neurons. Two-color immunoperoxidase staining for YFP (black) and ChAT (brown) in transverse sections through the thoracic and lumbar spinal cord. (A–C) At all segmental levels, lamina IX contains a moderate number of black varicose YFP-immunoreactive axons. Micrographs in montages: A, 3; B and C, 5. Scale bars: A and B, 100 μm; C, 250 μm. (D–I) Nevertheless, only rare, brown ChAT-immunoreactive somatic motor neurons receive close appositions (arrows) from black YFP-immunoreactive varicosities. Appositions from YFP-immunoreactive boutons do occur on the dendrites of somatic motor neurons (C–E). However, in most cases, YFP-containing varicosities appose the cell bodies of somatic motor neurons (F–I). LE, lumbar enlargement. Micrographs in montages: D-F & H, 3; G, 2; I, 5. Scale bars: D, 50 μm; E–I, 20 μm. (For interpretation of the references to color in this figure legend, the reader is referred to the web version of this article.)

**Fig. 8:**
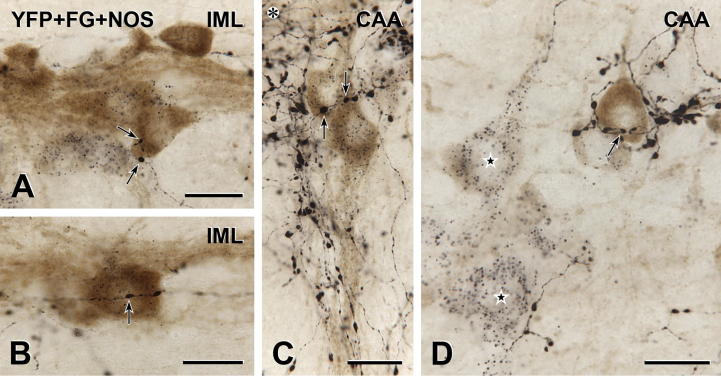
YFP-immunoreactive innervation of nitric oxide synthase (NOS)-immunoreactive spinal neurons. Triple immunoperoxidase staining for YFP (black), intraperitoneal FG (black) and NOS (brown) in the IML and CAA. Neurons containing FG-immunoreactivity send axons to the periphery and neurons without FG have axons that stay within the CNS. Hence, FG staining distinguishes between NOS-positive interneurons and NOS-positive SPN. Both NOS-containing SPN and NOS-containing spinal interneurons receive input from YFP-immunoreactive axons. (A and B) In the IML, SPN showing immunoreactivity for NOS (brown) and FG (black puncta within the cytoplasm) receive close appositions (arrows) from black YFP-immunoreactive boutons. A, Montage of 2 micrographs. Scale bars: 20 μm. (C) Similarly in the CAA, black YFP-immunoreactive varicosities closely appose (arrows) neurons that contain both brown NOS- and black FG-immunoreactivity, indicating that they are SPN. Asterisk, Ependymal cells lining the dorsal surface of the central canal are out of focus. Montage of 2 micrographs. Scale bar: 20 μm. (D) In the CAA, a varicosity from a black YFP-positive axon closely apposes (arrow) a brown NOS-positive neuron that lacks FG-immunoreactivity, indicating that it is an interneuron. Nearby neurons (stars) contain black FG-immunoreactive puncta, indicating that they are SPN. The top starred SPN is faintly immunoreactive for NOS. Montage of 5 micrographs. Scale bar: 20 μm. (For interpretation of the references to color in this figure legend, the reader is referred to the web version of this article.)

**Fig. 9:**
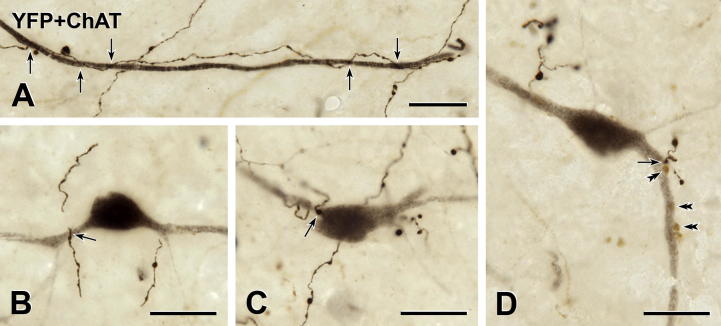
Innervation of spinal YFP-immunoreactive neurons. Two-color immunoperoxidase staining for YFP (black) and ChAT (brown) in transverse sections through the lumbar enlargement. (A) A YFP-immunoreactive axon forms several close appositions (arrows) on the dendrite of the YFP-immunoreactive spinal neuron shown in Fig. 2B. (B) A YFP-immunoreactive varicosity apposes (arrow) the proximal dendrite of a YFP-immunoreactive spinal neuron. (C) The cell body of a YFP-immunoreactive spinal neuron receives a close apposition (arrow) from a YFP-immunoreactive varicosity. (D) The dendrite of a YFP-positive spinal neuron receives a close apposition from a YFP-positive bouton (arrow) as well as from several ChAT-positive boutons (double arrowheads). Micrographs in montages: A, 12; D, 3. Scale bars: 20 μm. (For interpretation of the references to color in this figure legend, the reader is referred to the web version of this article.)

Varicose YFP-containing axons had clearly delineated varicosities and fine intervaricose segments (Figs. 4–9). Some of the axons had large varicosities; others had small fine varicosities and some axons had both large and small varicosities (e.g., Figs. 6B, C and 7E). All three types of axons made close appositions on immunohistochemically identified spinal neurons. In segments T1–L2 (Figs. 4 and 5), which contain SPN, many varicose, YFP-immunoreactive axons traveled rostrocaudally along the IML and mediolaterally between the IML and the intercalated nucleus (ICN). There was also a dense plexus of varicose axons containing YFP-immunoreactivity that ran rostrocaudally through the dorsal portion of lamina X, the location of SPN cell bodies in the spinal autonomic subnucleus called the central autonomic area (CAA). Smaller numbers of varicose YFP-positive axons occurred through lamina VII and in laminae VIII and IX; areas containing limb motor neurons (Figs. 4D and 7). There were fewer immunoreactive axons in laminae IV and V than in laminae containing autonomic and somatic motor neurons (Fig. 4A, E). The number of YFP-containing axons decreased from lamina IV to lamina II, where there were almost no YFP-immunoreactive axons. YFP-positive axons were never found in lamina I.

### YFP-immunoreactive innervation of spinal cholinergic neurons

We assessed the YFP innervation of spinal cholinergic neurons using two-color immunoperoxidase labeling in transverse and horizontal sections from spinal segments T1–S4. YFP-immunoreactive axons were detected with anti-GFP and a black peroxidase reaction product. Cholinergic neurons were revealed by immunoreactivity for ChAT, the acetylcholine synthesizing enzyme, and a brown reaction product. ChAT-positive neurons in the lateral horn of spinal segments T1–L2 were classified as SPN. Their cell bodies were concentrated in the IML and in the CAA above the central canal (i.e., dorsal lamina X). The ICN, which lies between the IML and the CAA, also contained SPN somata and a few SPN cells bodies were present in the dorsolateral funiculus. ChAT-immunoreactive neurons located in the spinal parasympathetic nucleus in the lateral horn of the lower lumbar and upper sacral cord were classified as PPN. ChAT-immunoreactive neurons lying in the ventral horn at any spinal level were considered to be somatic motor neurons.

### Innervation of SPN

In both transverse (Fig. 4) and horizontal (Fig. 5) sections, it was clear that the YFP innervation of the CAA was considerably more dense than the YFP innervation of either the IML or the ICN. There were also YFP-immunoreactive axons in the dorsolateral and lateral funiculi (Figs. 4A–E and 5A, B). Many of the varicose YFP-immunoreactive axons in the IML and ICN in spinal segments T1–L2 closely apposed the cell bodies and proximal dendrites of ChAT-immunoreactive SPN (Fig. 4A, B, E and F). Similarly, varicose YFP-positive axons in dorsal lamina X often formed close appositions on ChAT-positive SPN perikarya in the CAA (Figs. 4G, H and 5D–F). In both the IML and CAA, some ChAT-positive neurons received more than one apposition from YFP-immunoreactive terminals. This was particularly the case for SPN that lay within dense arrays of YFP-positive axons (Fig. 4B, H).

### Innervation of PPN

In transverse sections through the parasympathetic nucleus in segments L6 and S1, it was difficult to find YFP-immunoreactive terminals that closely apposed ChAT-immunoreactive PPN somata. Close appositions were much easier to find in horizontal sections (Fig. 6) but appositions were still uncommon. In horizontal sections, it was possible for us to determine the proportion of the PPN population that received YFP innervation. We examined a total of 948 PPN in two mice. In one mouse, YFP-immunoreactive varicosities closely apposed 64 of 502 PPN (12.8%); in the other mouse, 55 of 446 PPN (12.3%) received YFP-positive close appositions. In all cases, only one YFP-containing terminal apposed each innervated PPN.

### Innervation of somatic motor neurons

In transverse sections of T1–S4, the vast majority of ChAT-immunoreactive motor neurons in the ventral horn lacked YFP innervation (Fig. 7) although YFP-immunoreactive boutons did form close appositions on the cell bodies of rare ChAT-positive motor neurons (Fig. 7F–I). Appositions were also found on motor neuronal dendrites (Fig. 7E) but such axodendritic appositions were even less common than axosomatic appositions.

### YFP-immunoreactive innervation of neurons that contain NOS

We have previously shown that in IML and CAA, both SPN and interneurons contain immunoreactivity for NOS, the enzyme that synthesizes nitric oxide (Hinrichs and Llewellyn-Smith, 2009). To determine whether YFP-PPG neurons innervated spinal interneurons as well as SPN, we retrogradely labeled the entire population of SPN via intraperitoneal injections of FG (Anderson and Edwards, 1994) and then used triple immunoperoxidase labeling to localize YFP (black), FG (black) and NOS (brown) in horizontal sections of spinal cord (Fig. 8). Neurons that expressed NOS only were taken to be interneurons whereas those expressing NOS plus FG were presumed to be SPN.

In the IML, many neurons were immunoreactive for NOS and FG or FG alone (i.e., NOS-positive and NOS-negative SPN, respectively) whereas neurons that were immunoreactive for NOS alone (i.e., NOS-positive interneurons) were uncommon. The CAA contained substantial populations of NOS/FG neurons, FG only neurons and NOS only neurons. In the IML, YFP-immunoreactive varicosities closely apposed NOS/FG neurons (Fig. 8A, B). In the CAA, both NOS/FG neurons (Fig. 8C) and NOS only neurons (Fig. 8D) received close appositions from YFP-positive terminals. We could not determine whether YFP-positive axons innervated FG only neurons because we could not define the boundaries of neurons that contained only black intracellular punctate labeling for FG (Fig. 8D).

### YFP-immunoreactive innervation of spinal YFP-immunoreactive neurons

In the LE, YFP-immunoreactive boutons formed close appositions on the somata or proximal dendrites of a subset of the spinal neurons that contained YFP (Fig. 9A–D). To assess the extent of this innervation, we counted YFP-immunoreactive neurons that were and were not apposed by YFP-immunoreactive axons in transverse sections that were stained for YFP and either ChAT or NOS from seven YFP-PPG mice. Of the 195 YFP-expressing spinal neurons examined, 40 (20.5%) received close appositions from YFP-immunoreactive terminals.

Because the sections assessed for YFP appositions were double-stained for YFP plus a neurotransmitter-related marker, we were able to determine whether or not YFP-expressing spinal neurons received innervation from ChAT- or NOS-immunoreactive axons. ChAT-positive boutons closely apposed 23% (32 of 139) of the YFP-immunoreactive spinal neurons in sections stained for YFP plus ChAT with many of the innervated neurons receiving several appositions from ChAT-positive terminals (Figs. 9D). YFP-immunoreactive boutons also apposed five of the 32 YFP-immunoreactive spinal neurons with ChAT innervation (Fig. 9D). In contrast, none of the 56 YFP-immunoreactive spinal neurons in sections stained for YFP plus NOS were closely apposed by NOS-positive boutons.

### Source of spinal PPG innervation

**Fig. 10:**
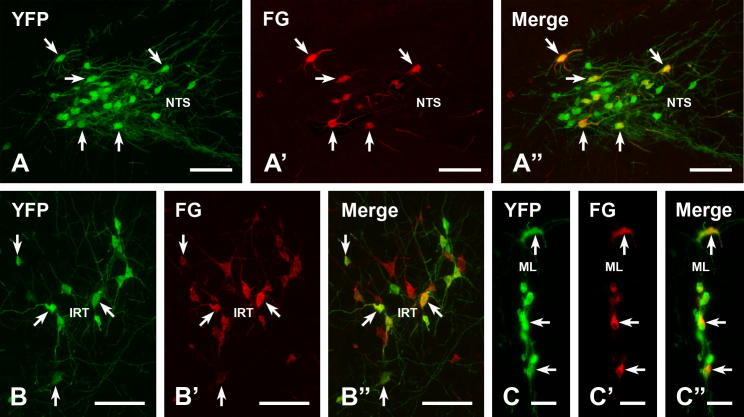
Spinally projecting YFP-PPG neurons in the brainstem. Double immunofluorescent staining for YFP (green) and FG (red) in the brainstems of YFP-PPG mice that received injections of FG at spinal segment T9. The NTS (A–A”), the IRT (B–B”) and dorsal midline (C–C”) contain YFP-immunofluorescent neurons (A–C), some of which contain FG-immunoreactivity (A’, B’ and C’). Double labeled neurons are spinally projecting YFP-PPG neurons. A”, B”, C”, merged images of micrographs showing YFP- and FG-immunoreactive neurons in each area. Scale bars: A, B, 100 μm; C, 50 μm. (For interpretation of the references to color in this figure legend, the reader is referred to the web version of this article.)

As reported previously (Llewellyn-Smith et al., 2011, 2013), we observed YFP-immunoreactive cell bodies in the caudal NTS and the medial portion of the IRT and in the midline (Fig. 10). In the caudal NTS, YFP-PPG somata extended from caudal to the caudal tip of the area postrema (AP) to roughly mid-AP level. At the caudal end of their distribution within the NTS, the YFP-PPG neurons were located medially whereas at the rostral end, they were mainly found laterally. Within the IRT, YFP-immunoreactive somata occurred from about mid-AP level to roughly where the NTS moved away from the fourth ventricle in a location that is dorsomedial to the nucleus ambiguus and the A1 cell group. In the midline, a few YFP-immunoreactive somata lay just ventral to the hypoglossal nucleus in the region equivalent to raphé obscurus (Fig. 10).

In mice with injections of the retrograde tracer FG into spinal segment T9, we found that many of the green YFP-immunofluorescent neurons in the brainstem also contained red FG immunofluorescence, indicating that their axons projected to the spinal cord. YFP-PPG neuron containing FG-immunoreactivity occurred not only in the NTS (Fig. 7A–A”) and IRT (Fig. 7B–B”) but also in the midline (Fig. 7C–C”).

We examined 614 medullary YFP-PPG neurons (416 NTS neurons, 149 IRT neurons and 49 midline neurons) in three mice for the presence of FG retrogradely transported from the spinal cord. Quantification of neurons double-stained for YFP and FG and single-stained for YFP showed that, overall, 353 of the 614 (57.5%) medullary YFP-PPG neurons contained FG-immunoreactivity. YFP-immunoreactive/FG-immunoreactive neurons occurred in all medullary regions that contained YFP-PPG neurons. Immunoreactivity for FG was found in 53.8 ± 10.6% of YFP-immunoreactive neurons in the NTS (231/416 neurons), 61.5 ± 6.3% of YFP-PPG neurons in the IRT (96/149 neurons) and 53.1 ± 4.3% of YFP-PPG neurons in the midline (26/49 neurons).

## Discussion

Here, we assessed the PPG innervation of neurochemically identified spinal neurons in YFP-PPG mice (Reimann et al., 2008; Llewellyn-Smith et al., 2011). In these mice YFP is expressed selectively in cells where the glucagon promoter is active (Reimann et al., 2008; Hisadome et al., 2010). Consequently, these mice show YFP-immunoreactivity exclusively in pancreatic α-cells that produce glucagon and enteroendocrine L-cells and in populations of central neurons that produce GLP-1 and GLP-2 (Reimann et al., 2008). YFP-expressing neurons in YFP-PPG mice produce PPG mRNA, as demonstrated by single-cell RT-PCR for YFP-PPG neurons in the NTS (Hisadome et al., 2010, 2011), and should therefore synthesize GLP-1 and GLP-2 in addition to YFP.

### Descending projections from brainstem PPG neurons

Previous studies have demonstrated that GLP-1-immunoreactive neurons located in the caudal NTS and the IRT send ascending projections to forebrain targets, including the DMH and the PVN (Vrang et al., 2007), as well as the ventral tegmental area and the nucleus accumbens (Dossat et al., 2011; Alhadeff et al., 2012). PPG neurons in the medulla also project locally to cholinergic and catecholaminergic neurons in the NTS and the DMV and to monoaminergic neurons in the ventral medulla (Llewellyn-Smith et al., 2013). However, little is known about descending GLP-1 projections to the spinal cord (Maniscalco et al., 2012).

The results of this study show for the first time that the spinal cords of YFP-PPG mice receive a substantial PPG innervation. Similar to our previous observations in the brain, we found that the highest densities of varicose YFP-immunoreactive axons occurred in some of the autonomic areas of the spinal cord. Specifically, spinal YFP innervation was preferentially targeted toward the CAA (i.e., dorsal lamina X) and the IML, areas that contain the cell bodies of SPN. In contrast, the sacral parasympathetic nucleus, which contains the cell bodies of PPN, was sparsely innervated. YFP-positive axons were moderately dense in laminae V, VIII and IX. The density of YFP innervation declined from lamina V to lamina II and we never observed a YFP-containing axon in lamina I. This distribution of YFP-PPG axons matches the distribution of neurons expressing the GLP-1 receptor in rat spinal cord (Merchenthaler et al., 1999), which were located in the ventral horn but not the dorsal horn (Merchenthaler et al., 1999).

The results of our retrograde tracing from spinal segment T9 have demonstrated that a substantial fraction of brainstem YFP-PPG neurons send axons to the spinal cord. Similar to PPG projections to the hypothalamus (Vrang et al., 2007), we found that YFP-PPG neurons located in the NTS, IRT and midline all projected to the spinal cord, suggesting there is no spatial segregation within the medulla of PPG neurons that innervate different targets. We have shown here that at least half of YFP-PPG neurons in the medulla have descending axons that reach the lower thoracic spinal cord. Sizeable proportions of PPG neurons are also known to project to hypothalamic sites (Vrang et al., 2007), to the nucleus accumbens (Dossat et al., 2011; Alhadeff et al., 2012) and to the ventral tegmental area (Alhadeff et al., 2012). Taken together, these observations suggest that individual PPG neurons might project to more than one target.

### YFP-immunoreactive neurons in the LE

One of the most surprising findings of this study was the discovery of a small, distinct population of YFP-immunoreactive neurons in the deep dorsal horn of the lower lumbar spinal cord. Unlike the PPG neurons in the NTS, we are currently unable to access these neurons for single-cell RT-PCR analysis *in vitro*. Thus, we cannot entirely exclude the possibility that these neurons express the YFP transgene ectopically, a problem that occasionally occurs in transgenic animals. Nevertheless, performing RT-PCR on RNA isolated from lumbosacral spinal cord allowed us to demonstrate the presence of PPG mRNA in this spinal region (Trapp, Manton, Edwards, unpublished observations). This finding supports the hypothesis that spinal YFP-PPG neurons do, in fact, produce GLP-1 although it is conceivably possible that the PCR signal might be due to mRNA in axons originating from YFP-PPG neurons in the NTS.

At present, the functional role of the YFP-immunoreactive neurons in the LE is unclear. Our injections of FG into T9 clearly show that these spinal YFP-positive neurons do not have axons that travel through the lower thoracic cord. Hence, the spinal YFP-synthesizing neurons do not project rostrally and therefore do not provide any of the innervation that we have previously described in the brain (Llewellyn-Smith et al., 2011, 2013). When we applied FG intraperitoneally, intraspinal YFP-immunoreactive neurons also failed to label. Hence, the axons of these neurons do not leave the spinal cord so their innervation targets must be within the CNS. It is possible that the spinal neurons expressing YFP project locally, possibly to PPN or to spinal interneurons that are part of spinal parasympathetic circuitry. Our experiments did not explore these possibilities.

### PPG innervation of ChAT and NOS immunoreactive spinal neurons

The spinal cord contains three major populations of ChAT-immunoreactive cholinergic neurons. These are SPN, PPN and somatic motor neurons. Our current study has demonstrated that, of these three functionally distinct groups of spinal neurons, SPN receive by far the highest density of close appositions from varicosities of YFP-PPG axons. We also found that the CAA, which contains many of the SPN projecting to the celiac, inferior mesenteric and major pelvic ganglia (Fenwick et al., 2006; Hinrichs and Llewellyn-Smith, 2009), was more heavily innervated by YFP-immunoreactive axons than the IML. Unlike SPN, PPN in the sacral parasympathetic nucleus received a meagre GLP-1 innervation, with YFP-PPG axons closely apposing only about 10% of these neurons. The GLP-1 input to somatic motor neurons was even sparser; only rare ChAT-positive neurons throughout the rostrocaudal extent of the ventral horn were closely apposed by YFP-immunoreactive axons. We have assumed that close appositions from YFP-containing axon terminals signify innervation. While this assumption could only be confirmed with electron microscopy, ultrastructural studies on other central autonomic neurons by Pilowsky et al. (1992) have verified that at least half of the terminals that form close appositions at the light microscope level form synaptic contacts at the ultrastructural level. Furthermore, electron microscopy has recently confirmed that GLP-1 neurons form synapses in the CNS with the demonstration of synapses from GLP-1-immunoreactive axon terminals onto dendrites in rat hypothalamus (Zheng et al., 2014).

NOS-immunoreactive interneurons also occur in the CAA (Hinrichs and Llewellyn-Smith, 2009) so we investigated whether CAA interneurons received PPG innervation. In mice with intraperitoneal FG injections, which are known to label all SPN (Anderson and Edwards, 1994), we classified NOS-immunoreactive neurons in the CAA as interneurons if they lacked FG-immunostaining. We found that many of the NOS-positive/FG-negative CAA neurons received close appositions from YFP-immunoreactive axons, indicating that spinal interneurons as well as SPN are targeted by the spinal axons of brainstem PPG neurons.

Taken together, our observations show that, in the spinal cord, PPG innervation is preferentially directed toward SPN, with SPN in the CAA being more heavily innervated than SPN in the IML or ICN. In contrast, PPG axons only lightly innervate the spinal parasympathetic preganglionic population. Whether PPG axons target a specific functional subset of PPN will require experiments using transynaptic viral labeling or functional neuroanatomical approaches. ChAT-positive neurons in the ventral horn received by far the lightest PPG input. This input is so miniscule that activation of brainstem PPG neurons is very unlikely to affect the activity of somatic motor neurons.

### Central PPG neurons in the control of autonomic functions

Neurons in the NTS are generally thought to influence sympathetic outflow indirectly through a relay in the ventral medulla or through ascending projections to hypothalamic nuclei that provide direct input to SPN in the spinal cord. Although medullary PPG neurons might be part of such bulbospinal or hypothalamic-spinal pathways (Llewellyn-Smith et al., 2011, 2013), the present study demonstrates that there is a substantial direct projection from PPG neurons in the NTS, IRT and midline to spinal sympathetic nuclei. Spinally projecting neurons in the NTS and IRT have been reported in the rat (Mtui et al., 1993). The striking similarity in the distribution of NTS and IRT neurons projecting to the spinal cord in that study with the distribution of spinally projecting YFP-PPG neurons found here suggests that PPG neurons might constitute a sizeable fraction of the NTS and IRT neurons that supply axons to the spinal cord. Furthermore, a good proportion of the spinally projecting NTS neurons found by Mtui et al. (1993) had terminals in the CAA in addition to the IML, similar to our observations here.

### Gastrointestinal function

The present study has demonstrated a dense PPG innervation of SPN, particularly SPN whose cell bodies lie in the CAA. The CAA contains many SPN that project to prevertebral ganglia, including the celiac, mesenteric and major pelvic ganglia (Fenwick et al., 2006; Hinrichs and Llewellyn-Smith, 2009). Since sympathetic post-ganglionic neurons in these ganglia innervate the entirety of the gastrointestinal tract from the stomach to the rectum (Keast and de Groat, 1989; Dhami and Mitchell, 1994; Domoto and Tsumori, 1994; Trudrung et al., 1994; Quinson et al., 2001), GLP-1 input to SPN could modulate sympathetic outflow to every section of the gut. The celiac and superior mesenteric ganglia have been shown to contain SPN that are part of the circuitry controlling splanchnic blood vessels (Macrae et al., 1986; Hsieh et al., 2000). If PPG axons target SPN in circuits controlling the mesenteric vasculature, then PPG neurons in the medulla could influence not only motility, secretion and absorption in the gut but also its blood supply.

### Cardiovascular regulation

In addition to a role in regulating gut function, brainstem PPG neurons may also affect cardiovascular control. Anatomical findings from this and previous studies suggest that PPG neurons could modulate sympathetic outflow via descending, ascending and/or intramedullary pathways (Rinaman, 1999; Vrang et al., 2007; Tauchi et al., 2008; Llewellyn-Smith et al., 2011, 2013; Gu et al., 2013). When injected intracerebroventricularly (i.c.v.), the GLP-1 receptor agonist exendin-4 (Ex-4) raises blood pressure and heart rate (Yamamoto et al., 2002; Cabou et al., 2008; Hayes et al., 2008; Griffioen et al., 2011). The tachycardia and hypertension in response to 4th ventricular Ex-4 injection persist in chronic decerebrate rats so hypothalamic GLP-1 receptors are not necessary for the cardiovascular responses (Hayes et al., 2008). However, it is not known whether i.c.v. Ex-4 reaches GLP-1 receptors located on SPN in the CAA or IML or CAA interneurons or whether intrathecal injections of Ex-4 have similar cardiovascular effects to i.c.v. injections. Studies addressing these questions would help to define the cardiovascular actions of centrally-administered GLP-1.

### Thermoregulation

Central GLP-1 can also affect thermoregulatory responses. However, both the pathways involved and the direction of the effect are still under discussion. A number of studies have reported hypothermic effects of GLP-1 receptor agonists injected into the brain. For instance, Hayes et al. (2008) reported Ex-4 injection into the fourth ventricle caused hypothermia in rat. Similarly, blockade of hindbrain GLP-1 receptor with exendin-9 prevents CART-induced hypothermia (Skibicka et al., 2009). Conversely, Osaka et al. (2005) observed an increase in core temperature in response to GLP-1; and Lockie and colleagues (2012) reported that i.c.v. infusion of GLP-1 caused an increase in temperature in brown adipose tissue in mice. We have previously described PPG innervation of serotonin synthesizing neurons in raphé pallidus (Llewellyn-Smith et al., 2013). Because serotonin neurons in this region are part of the circuitry that activates SPN in the spinal cord to promote heat production from brown adipose tissue (Morrison et al., 2008), a GLP-1 input to raphé-spinal 5-HT neuron could be the link between central PPG neurons and serotonin dependent thermogenesis. In addition, this study suggests that medullary PPG neurons might influence thermoregulation at the spinal cord level. Injection of pseudorabies virus into intrascapular brown fat transynaptically labels SPN in the IML of upper thoracic cord (Oldfield et al., 2002; Cano et al., 2003) and we have shown here that SPN in this region receive close appositions from the axons of brainstem YFP-PPG neurons. Hence, it is possible that GLP-1 may play a role in thermoregulation at the spinal cord level. Studies involving either intrathecal injections of GLP-1 agonists and antagonists or activation of PPG neurons in the brainstem to release GLP-1 in the spinal cord would clarify this issue.

Electrophysiological and viral tracing studies have identified spinal sympathetic interneurons in the CAA (Tang et al., 2004; Deuchars et al., 2005) and we have shown here that there is PPG innervation of CAA interneurons. If the population of spinal sympathetic interneurons identified by Tang et al. (2004) and Deuchars et al. (2005) includes NOS interneurons in the CAA, then brainstem PPG neurons may also influence sympathetic function indirectly through inputs to spinal interneurons as well as directly through input to SPN.

### Parasympathetic function

In contrast to spinal sympathetic regions, we found that PPG innervation of PPN in the parasympathetic nucleus in lumbosacral cord was sparse, with PPG terminals closely apposing only about 10% of these neurons. GLP-1 has significant effects on vagal function (Holmes et al., 2009; Griffioen et al., 2011) through actions on cranial PPN and it would be interesting to determine whether there are effects on one or more functional types of sacral PPN. Trans-synaptic viral tracing would identify whether GLP-1 input to sacral PPN could influence the control of one or more of the pelvic organs that are the innervation targets of the parasympathetic post-ganglionic neurons to which sacral PPN provide input. In particular, it would be interesting to know whether PPG innervation in the sacral parasympathetic nucleus is directed toward PPN in pathways controlling the lower bowel.

All of these observations suggest that brainstem PPG neurons could directly modulate sympathetic outflow to a variety of target organs through spinal inputs to SPN. PPG neurons in the medulla could also have an indirect effect on sympathetic outflow through their innervation of interneurons in the CAA. Given that PPG neurons seem to affect both sympathetic and parasympathetic outflows, it is pertinent to investigate whether GLP-1 globally activates one outflow and inhibits the other, i.e., the effects are synergistic. Alternatively, GLP-1 effects on sympathetic and parasympathetic outflows could be independent and locally focussed.

## Conclusions

In summary, this study provides the first detailed description of the PPG innervation of neurochemically identified neurons in the spinal cord. Our results suggest that spinal sympathetic circuits are a main innervation target of PPG neurons in the brainstem, which provide strong innervation to both SPN and spinal interneurons that may be involved in regulating sympathetic function. We also found that more than half of the medullary PPG neurons are spinally-projecting. Finally, we discovered a population of neurons in the deep dorsal horn of the lower lumbar spinal cord that expressed YFP and may be capable of synthesizing and releasing GLP-1. The function(s) of these spinal YFP-immunoreactive neurons is not clear but they do not provide any innervation to the brain. Our observations add another level of complexity to the modulation of autonomic outflow by central PPG neurons.

## Figures and Tables

**Table 1:** Antibodies used

Antigen	Immunogen	Manufacturer, species antibody was raised in, mono- vs. polyclonal, catalog and lot number	Dilution used
*Primary antibodies*			
Green fluorescent protein (GFP)	Recombinant full-length green fluorescent protein	Abcam (Cambridge, MA, USA), chicken polyclonal, catalog # AB13970, lot # 623923	1:10,000^b^ 1:50,000^a^ 1:100,000^a^

Choline acetyltransferase (ChAT)	Human placental choline acetyltransferase	Chemicon (Temecula, CA, USA), goat polyclonal, catalog # AB144P, lot # 18110644 or JC1669317	1:5,000^a^

Neuronal nitric oxide synthase (NOS)	C-terminal synthetic peptide sequence corresponding to amino acids 1419–1433 of human neuronal NOS coupled to keyhole limpet hemocyanin	ImmunoStar (Stillwater MN, USA), rabbit polyclonal, catalog # 24287, lot # 043005	1:50,000^a^ 1:150,000^a^

Fluorogold (FG)	Glutaraldehyde conjugate of Fluorogold	Protos Biotech Corporation (New York, NY, USA), guinea-pig polyclonal, catalog # NM-01; no lot number provided	1:2,000^b^ 1:20,000^a^ 1:50,000^a^

*Secondary antibodies*			
Biotinylated donkey anti-chicken IgY		Catalog # 703-065-155; Jackson ImmunoResearch (West Grove, PA, USA)	1:500
Biotinylated donkey anti-goat IgG		Catalog # 705-065-147; Jackson ImmunoResearch (West Grove, PA, USA)	1:500
Biotinylated donkey anti-rabbit IgG		Catalog # 711-065-152; Jackson ImmunoResearch (West Grove, PA, USA)	1:500
Biotinylated donkey anti-guinea-pig IgG		Catalog # 705-065-147; Jackson ImmunoResearch (West Grove, PA, USA)	1:500
FITC-donkey anti-guinea-pig IgG		Catalog # 706-095-148; Jackson ImmunoResearch (West Grove, PA, USA)	1:200

a Immunoperoxidase staining.

b Immunofluorescent staining.

## References

[b0005] Alhadeff A.L., Rupprecht L.E., Hayes M.R. (2012). GLP-1 neurons in the nucleus of the solitary tract project directly to the ventral tegmental area and nucleus accumbens to control for food intake. Endocrinology.

[b0010] Anderson C.R., Edwards S.L. (1994). Intraperitoneal injections of Fluorogold reliably labels all sympathetic preganglionic neurons in the rat. J Neurosci Methods.

[b0015] Barrera J.G., Jones K.R., Herman J.P., D’Alessio D.A., Woods S.C., Seeley R.J. (2011). Hyperphagia and increased fat accumulation in two models of chronic CNS glucagon-like peptide-1 loss of function. J Neurosci.

[b0020] Buse J.B., Rosenstock J., Sesti G., Schmidt W.E., Montanya E., Brett J.H., Zychma M., Blonde L. (2009). Liraglutide once a day versus exenatide twice a day for type 2 diabetes: a 26-week randomised, parallel-group, multinational, open-label trial (LEAD-6). Lancet.

[b0025] Cabou C., Campistron G., Marsollier N., Leloup C., Cruciani-Guglielmacci C., Penicaud L., Drucker D.J., Magnan C., Burcelin R. (2008). Brain glucagon-like peptide-1 regulates arterial blood flow, heart rate, and insulin sensitivity. Diabetes.

[b0030] Cano G., Passerin A.M., Schiltz J.C., Card J.P., Morrison S.F., Sved A.F. (2003). Anatomical substrates for the central control of sympathetic outflow to interscapular adipose tissue during cold exposure. J Comp Neurol.

[b0035] Deuchars S.A., Milligan C.J., Stornetta R.L., Deuchars J. (2005). GABAergic neurons in the central region of the spinal cord: a novel substrate for sympathetic inhibition. J Neurosci.

[b0040] Dhami D., Mitchell B.S. (1994). Chemical coding of neurons projecting to pelvic viscera in the male guinea pig: a study by retrograde transport and immunohistochemistry. Histochem J.

[b0045] Domoto T., Tsumori T. (1994). Co-localization of nitric oxide synthase and vasoactive intestinal peptide immunoreactivity in neurons of the major pelvic ganglion projecting to the rat rectum and penis. Cell Tissue Res.

[b0050] Dossat A.M., Lilly N., Kay K., Williams D.L. (2011). Glucagon-like peptide 1 receptors in nucleus accumbens affect food intake. J Neurosci.

[b0055] Drucker D.J., Nauck M.A. (2006). The incretin system: glucagon-like peptide-1 receptor agonists and dipeptidyl peptidase-4 inhibitors in type 2 diabetes. Lancet.

[b0060] Fenwick N.M., Martin C.L., Llewellyn-Smith I.J. (2006). Immunoreactivity for cocaine- and amphetamine-regulated transcript in rat sympathetic preganglionic neurons projecting to sympathetic ganglia and the adrenal medulla. J Comp Neurol.

[b0065] Griffioen K.J., Wan R., Okun E., Wang X., Lovett-Barr M.R., Li Y., Mughal M.R., Mendelowitz D., Mattson M.P. (2011). GLP-1 receptor stimulation depresses heart rate variability and inhibits neurotransmission to cardiac vagal neurons. Cardiovasc Res.

[b0070] Gu G., Roland B., Tomaselli K., Dolman C.S., Lowe C., Heilig J.S. (2013). Glucagon-like peptide-1 in the rat brain: distribution of expression and functional implication. J Comp Neurol.

[b0075] Hayes M.R., Bradley L., Grill H.J. (2009). Endogenous hindbrain glucagon-like peptide-1 receptor activation contributes to the control of food intake by mediating gastric satiation signaling. Endocrinology.

[b0080] Hayes M.R., Skibicka K.P., Grill H.J. (2008). Caudal brainstem processing is sufficient for behavioral, sympathetic, and parasympathetic responses driven by peripheral and hindbrain glucagon-like-peptide-1 receptor stimulation. Endocrinology.

[b0085] Hinrichs J.M., Llewellyn-Smith I.J. (2009). Variability in the occurrence of nitric oxide synthase immunoreactivity in different populations of rat sympathetic preganglionic neurons. J Comp Neurol.

[b0090] Hisadome K., Reimann F., Gribble F.M., Trapp S. (2010). Leptin directly depolarizes preproglucagon neurons in the nucleus tractus solitarius: electrical properties of glucagon-like Peptide 1 neurons. Diabetes.

[b0095] Hisadome K., Reimann F., Gribble F.M., Trapp S. (2011). CCK stimulation of GLP-1 neurons involves {alpha}1-adrenoceptor-mediated increase in glutamatergic synaptic inputs. Diabetes.

[b0100] Holmes G.M., Browning K.N., Tong M., Qualls-Creekmore E., Travagli R.A. (2009). Vagally mediated effects of glucagon-like peptide 1: in vitro and in vivo gastric actions. J Physiol.

[b0105] Holst J.J. (2007). The physiology of glucagon-like peptide 1. Physiol Rev.

[b0110] Holst J.J. (2013). Incretin hormones and the satiation signal. Int J Obes (Lond).

[b0115] Hsieh N.K., Liu J.C., Chen H.I. (2000). Localization of sympathetic postganglionic neurons innervating mesenteric artery and vein in rats. J Auton Nerv Syst.

[b0120] Jin S.L., Han V.K., Simmons J.G., Towle A.C., Lauder J.M., Lund P.K. (1988). Distribution of glucagonlike peptide I (GLP-I), glucagon, and glicentin in the rat brain: an immunocytochemical study. J Comp Neurol.

[b0125] Keast J.R., de Groat W.C. (1989). Immunohistochemical characterization of pelvic neurons which project to the bladder, colon, or penis in rats. J Comp Neurol.

[b0130] Larsen P.J., Tang-Christensen M., Holst J.J., Orskov C. (1997). Distribution of glucagon-like peptide-1 and other preproglucagon-derived peptides in the rat hypothalamus and brainstem. Neuroscience.

[b0135] Llewellyn-Smith I.J., Dicarlo S.E., Collins H.L., Keast J.R. (2005). Enkephalin-immunoreactive interneurons extensively innervate sympathetic preganglionic neurons regulating the pelvic viscera. J Comp Neurol.

[b0140] Llewellyn-Smith I.J., Gnanamanickam G.J., Reimann F., Gribble F.M., Trapp S. (2013). Preproglucagon (PPG) neurons innervate neurochemically identified autonomic neurons in the mouse brainstem. Neuroscience.

[b0145] Llewellyn-Smith I.J., Martin C.L., Fenwick N.M., Dicarlo S.E., Lujan H.L., Schreihofer A.M. (2007). VGLUT1 and VGLUT2 innervation in autonomic regions of intact and transected rat spinal cord. J Comp Neurol.

[b0150] Llewellyn-Smith I.J., Reimann F., Gribble F.M., Trapp S. (2011). Preproglucagon neurons project widely to autonomic control areas in the mouse brain. Neuroscience.

[b0155] Lockie S.H., Heppner K.M., Chaudhary N., Chabenne J.R., Morgan D.A., Veyrat-Durebex C., Ananthakrishnan G., Rohner-Jeanrenaud F., Drucker D.J., DiMarchi R., Rahmouni K., Oldfield B.J., Tschop M.H., Perez-Tilve D. (2012). Direct control of brown adipose tissue thermogenesis by central nervous system glucagon-like peptide-1 receptor signaling. Diabetes.

[b0160] Macrae I.M., Furness J.B., Costa M. (1986). Distribution of subgroups of noradrenaline neurons in the coeliac ganglion of the guinea-pig. Cell Tissue Res.

[b0165] Maniscalco J.W., Kreisler A.D., Rinaman L. (2012). Satiation and stress-induced hypophagia: examining the role of hindbrain neurons expressing prolactin-releasing Peptide or glucagon-like Peptide 1. Front Neurosci.

[b0170] Merchenthaler I., Lane M., Shughrue P. (1999). Distribution of pre-pro-glucagon and glucagon-like peptide-1 receptor messenger RNAs in the rat central nervous system. J Comp Neurol.

[b0175] Morrison S.F., Nakamura K., Madden C.J. (2008). Central control of thermogenesis in mammals. Exp Physiol.

[b0180] Mtui E.P., Anwar M., Gomez R., Reis D.J., Ruggiero D.A. (1993). Projections from the nucleus tractus solitarii to the spinal cord. J Comp Neurol.

[b0185] Oldfield B.J., Giles M.E., Watson A., Anderson C., Colvill L.M., McKinley M.J. (2002). The neurochemical characterisation of hypothalamic pathways projecting polysynaptically to brown adipose tissue in the rat. Neuroscience.

[b0190] Osaka T., Endo M., Yamakawa M., Inoue S. (2005). Energy expenditure by intravenous administration of glucagon-like peptide-1 mediated by the lower brainstem and sympathoadrenal system. Peptides.

[b0195] Pilowsky P., Llewellyn-Smith I.J., Lipski J., Chalmers J. (1992). Substance P immunoreactive boutons form synapses with feline sympathetic preganglionic neurons. J Comp Neurol.

[b0200] Quinson N., Robbins H.L., Clark M.J., Furness J.B. (2001). Locations and innervation of cell bodies of sympathetic neurons projecting to the gastrointestinal tract in the rat. Arch Histol Cytol.

[b0205] Reimann F., Habib A.M., Tolhurst G., Parker H.E., Rogers G.J., Gribble F.M. (2008). Glucose sensing in L cells: a primary cell study. Cell Metab.

[b0210] Rinaman L. (1999). Interoceptive stress activates glucagon-like peptide-1 neurons that project to the hypothalamus. Am J Physiol.

[b0215] Shughrue P.J., Lane M.V., Merchenthaler I. (1996). Glucagon-like peptide-1 receptor (GLP1-R) mRNA in the rat hypothalamus. Endocrinology.

[b0220] Skibicka K.P., Alhadeff A.L., Grill H.J. (2009). Hindbrain cocaine- and amphetamine-regulated transcript induces hypothermia mediated by GLP-1 receptors. J Neurosci.

[b0225] Tang X., Neckel N.D., Schramm L.P. (2004). Spinal interneurons infected by renal injection of pseudorabies virus in the rat. Brain Res.

[b0230] Tauchi M., Zhang R., D’Alessio D.A., Stern J.E., Herman J.P. (2008). Distribution of glucagon-like peptide-1 immunoreactivity in the hypothalamic paraventricular and supraoptic nuclei. J Chem Neuroanat.

[b0235] Trudrung P., Furness J.B., Pompolo S., Messenger J.P. (1994). Locations and chemistries of sympathetic nerve cells that project to the gastrointestinal tract and spleen. Arch. Histol. Cytol..

[b0240] Turton M.D., O’Shea D., Gunn I., Beak S.A., Edwards C.M., Meeran K., Choi S.J., Taylor G.M., Heath M.M., Lambert P.D., Wilding J.P., Smith D.M., Ghatei M.A., Herbert J., Bloom S.R. (1996). A role for glucagon-like peptide-1 in the central regulation of feeding. Nature.

[b0245] Vrang N., Hansen M., Larsen P.J., Tang-Christensen M. (2007). Characterization of brainstem preproglucagon projections to the paraventricular and dorsomedial hypothalamic nuclei. Brain Res.

[b0250] Williams D.L., Baskin D.G., Schwartz M.W. (2009). Evidence that intestinal glucagon-like peptide-1 plays a physiological role in satiety. Endocrinology.

[b0255] Yamamoto H., Lee C.E., Marcus J.N., Williams T.D., Overton J.M., Lopez M.E., Hollenberg A.N., Baggio L., Saper C.B., Drucker D.J., Elmquist J.K. (2002). Glucagon-like peptide-1 receptor stimulation increases blood pressure and heart rate and activates autonomic regulatory neurons. J Clin Invest.

[b0260] Zheng H., Stornetta R.L., Agassandian K., Rinaman L. (2014). Glutamatergic phenotype of glucagon-like peptide 1 neurons in the caudal nucleus of the solitary tract in rats. Brain Struct Funct.

